# Association between Vitamin D Receptor Single-Nucleotide Polymorphisms and Colorectal Cancer in the Thai Population: A Case-Control Study

**DOI:** 10.1155/2020/7562958

**Published:** 2020-06-15

**Authors:** Sirinporn Suksawatamnuay, Supachaya Sriphoosanaphan, Prapimphan Aumpansub, Satimai Aniwan, Kessarin Thanapirom, Suebpong Tanasanvimon, Panarat Thaimai, Sumitra Wiangngoen, Yuwadee Ponauthai, Sakolkan Sumdin, Pattama Angspatt, Rungsun Rerknimitr, Yong Poovorawan, Piyawat Komolmit

**Affiliations:** ^1^Division of Gastroenterology, Department of Medicine, Faculty of Medicine, Chulalongkorn University, Bangkok 10330, Thailand; ^2^Center of Excellence in Liver Diseases, King Chulalongkorn Memorial Hospital, Thai Red Cross Society, Bangkok 10330, Thailand; ^3^Liver Fibrosis and Cirrhosis Research Unit, Chulalongkorn University, Bangkok, Thailand; ^4^Division of Medical Oncology, Department of Medicine, Faculty of Medicine, Chulalongkorn University, Bangkok 10330, Thailand; ^5^Center of Excellence in Clinical Virology, Faculty of Medicine, Chulalongkorn University, Bangkok 10330, Thailand

## Abstract

Vitamin D and its cognate intracellular nuclear receptor, namely, vitamin D receptor (VDR), are involved in the regulation of a variety of body metabolic processes, immune function, and oncogenesis. A large number of studies demonstrated the association of low vitamin D levels and variations in five common single nucleotide polymorphisms (SNPs), *Fok*I, *Bsm*I, *Tru9*I, *Apa*I, and *Taq*I, with the risk of several cancers, including colorectal cancers. However, these associations vary among different populations. This case-control study was aimed at analysing whether common single-nucleotide polymorphisms (SNPs) and haplotypes of the vitamin D receptor (*VDR*) gene contribute to colorectal carcinogenesis in the Thai population. We enrolled 364 Thai participants from King Chulalongkorn Memorial Hospital between 2014 and 2015. Half of the participants underwent colonoscopy and showed a normal colon without polyps (control group) and another half were newly diagnosed patients with colorectal cancer (CRC) by colonoscopy during the index period, were under treatment, or were followed up at the outpatient clinic (case group). Differences in allele and genotype frequencies of five common *VDR* SNPs, between the case and control groups, were the primary outcome measures. Differences in haplotype frequencies of the five SNPs between the case and control groups were the secondary outcome measures. Among the 364 participants, baseline characteristics were not significantly different between the case and control groups, except for the higher proportion of males in the CRC group. The mean vitamin D level was also not significantly different between the case and control groups (24.6 ± 9.1 vs. 25.3 ± 10.6 ng/mL, *p* = 0.52). None of the five *VDR* SNPs was associated with CRC development (*p* > 0.05). However, haplotype analysis of these polymorphisms demonstrated that the AGGT haplotype was associated with a decreased risk of CRC (odds ratio 0.24, 95% confidence interval 0.07-0.81, *p* = 0.01). The AGGT haplotype was associated with a lower risk of CRC in the Thai population. This genetic linkage might support the role of vitamin D in colorectal carcinogenesis. However, this finding requires further study within a larger population and a multivariate analysis of other established risk factors.

## 1. Introduction

Colorectal cancer (CRC) is one of the most common malignancies worldwide. It is estimated that the incidence and burden of CRC will increase to 60% by 2030, especially in low-income and middle-income countries [1]. The prevention and early detection of CRC are therefore crucial to decrease morbidity and mortality. Besides the development of an effective strategy for prioritising a screening program based on patient risk, further study of CRC risk factors also has its benefits [2]. Vitamin D deficiency is one of the nutritional factors related to CRC risk. A previous epidemiologic study showed a higher prevalence of CRC in areas with low solar ultraviolet B exposure. Recent reviews, both *in vitro* and *in vivo*, also suggested an association between vitamin D deficiency and an increased risk of various malignancies, including breast cancer, prostate cancer, and CRC [3, 4]. Furthermore, maintaining a normal vitamin D level has been shown to reduce CRC risk [5–7].

Vitamin D is thought to affect cancer development via the cell-cycle processes, including cell proliferation, differentiation, and apoptosis, and through modification of immune activity [8, 9]. Vitamin D metabolism occurs primarily in the liver, kidney, and other tissues, including intestinal epithelial cells and immune cells. The active form of vitamin D exerts its effects through interaction with its cognate intracellular nuclear receptor, the vitamin D receptor (VDR), which subsequently activates a number of genes involved in immune cell proliferation, oncogenesis, and tumour suppression [9, 10].

The VDR gene is located on the long arm of chromosome 12 (12q13-14), spanning approximately 75 kb. It contains nine exons. Even though many SNPs associated with VDR function have been discovered, five common SNPs, namely, *Fok*I, *Bsm*I, *Tru9*I, *Apa*I, and *Taq*I, were mostly used in association studies, as they are usually coinherited as blocks of haplotypes [11]. Among the five SNPs, only *Fok*I is a nonsynonymous SNP located at the 5′ end of exon 2. The other four SNPs are the nonsynonymous variations. *Bsm*I, *Tru9*I, and *Apa*I are inside intron 8, and only *Taq*I is in exon 9, which is unlikely to affect the translated forms of VDR polypeptides [12]. Only the variation in *Fok*I genotypes (T>C allele) caused a change in the translation start site, which in turn resulted in a smaller protein with increased activity [13].

The downstream signals of the VDR involve different functions, including bone mineral metabolism, immune regulation, and cell growth and differentiation [14]. Studies of common polymorphisms in the *VDR* gene have demonstrated association of the polymorphisms with several chronic diseases such as systemic lupus erythematosus and osteoporosis and various malignancies, including CRC [15–18]. However, the previous data on the association between *VDR* polymorphisms and CRC risk were mostly from Western countries, and the collective results were inconsistent [15, 19]. These discrepancies might stem from the divergence of *VDR* allele frequencies among ethnic groups. As there is not enough data on the Thai population, this study was aimed at analysing whether common single-nucleotide polymorphisms (SNPs) and specific haplotypes of the *VDR* gene contribute to colorectal carcinogenesis in the Thai population.

## 2. Materials and Methods

### 2.1. Participants

This case-control study was conducted between 2014 and 2015 at King Chulalongkorn Memorial Hospital, a tertiary care centre in Bangkok, Thailand. The inclusion criteria for the CRC cases included the patients who were diagnosed at the time colonoscopy for CRC screening or those requiring investigation of the clinical cases for pathological confirmation. In addition, the CRC patients who were currently on follow-up treatment in the oncology clinic were also included in the study. The controlled cases were the patients who underwent colonoscopy examination for the routine CRC screening and showed normal colon without any type of colorectal polyps. In addition, the cases of patients who had current or previous diagnosis of inflammatory bowel diseases were excluded. The other exclusion criteria for both groups included having a history of other malignancies and declination to participate in the study. The study protocol was approved by the Institutional Review Board of the Faculty of Medicine of Chulalongkorn University (number 192/58). All participants provided written informed consent. We enrolled a total 364 Thai participants, of whom 182 underwent colonoscopy and showed a normal colon without any polyps (control group) and 182 had CRC (case group). The case group consisted of 90 patients who were newly diagnosed with CRC by colonoscopy during the index period and 92 patients who were undergoing treatment or were followed up at the outpatient clinic. All cases were diagnosed as positive malignancies based on pathological reports. Demographic variables, including age, sex, height, and weight, were recorded, and body mass index (BMI) was calculated. Each participant provided a blood sample collected in a 6 mL tube with ethylenediaminetetraacetic acid (EDTA) and stored at 4°C until analysis for vitamin D level and *VDR* genotype.

### 2.2. Vitamin D Assays

Vitamin D levels in the plasma samples were measured using the LIAISON® 25 OH Vitamin D TOTAL Assay (DiaSorin, Saluggia, Italy) and LIAISON® analyser, following the manufacturer's instructions. The final measurements were indicated in nanograms per millilitre.

### 2.3. Genotype Analysis

Genomic DNA was extracted from peripheral blood leukocytes in 6 mL of whole blood sample using standard phenol-chloroform protocols. Samples were sent to the lab consecutively in the order they were collected and genotyped by scientists who were blinded to the disease status of the patients in the study. Five SNPs of the *VDR* gene—namely, *Fok*I (rs228570 C>T), *Bsm*I (rs1544410 A>G), *Tru9*I (rs757343 G>A), *Apa*I (rs7975232 G>T), and *Taq*I (rs731236 C>T)—were genotyped using polymerase chain reaction-restriction fragment length polymorphism (PCR-RFLP) analysis. Human genomic DNA was extracted from 100 *μ*L of peripheral blood mononuclear cells by first incubating with proteinase K in a lysis buffer and then performing standard phenol-chloroform extraction and ethanol precipitation. The resultant pellet was dissolved in 50 *μ*L sterile water and stored at -20°C until analysis. Specific primer pairs for each locus of the *VDR* gene were used for amplification (Supplementary Table 1). Two microlitres of DNA was used to set up 25 *μ*L PCR reactions using the Perfect*Taq™* Plus Master Mix (5 Prime GmbH, Hamburg, Germany). A total volume of 10 *μ*L was digested overnight with 2 units of restriction endonucleases (New England Biolabs, Hitchin, UK) using the buffers and temperatures recommended by the manufacturer. The resultant DNA fragments were resolved by 2% agarose gel electrophoresis, and the RFLP result was visualized under ultraviolet light after staining with ethidium bromide. To validate the RFLP genotyping results, we selected the DNA product of each RFLP pattern in the 5 SNPs for DNA sequencing. Sanger sequencing of the DNA products was conducted using Macrogen (Seoul, Korea). All sequences were aligned with their reference sequence of the *VDR* gene obtained from GenBank (accession number NC_000012.12).

### 2.4. Statistical Methods

Statistical analyses were performed using SPSS software (version 20, 2015; SPSS Inc., Chicago, IL, USA). Numerical data are presented as means and standard deviations and categorical data as numbers and percentages. To compare associations between cases and controls, numerical and categorical variables were calculated using independent sample *t*-tests, chi-squared test, and Fisher's exact test, as appropriate. The normality of continuous variables was assessed by visually inspecting histograms and conducting a Shapiro-Wilk test. The reported *p* values were obtained from a two-sided statistical test, and a *p* value of <0.05 was considered statistically significant, except for logistic regression analyses where *p* values of 0.01 were considered significant based on a Bonferroni adjusted *p* with 5 comparisons. The Hardy-Weinberg equilibrium (HWE) of genotype frequencies among participants, linkage disequilibrium (LD), haplotype construction, and genetic association were analysed using SHEsis software [20].

Sample size was calculated for an unmatched case-control study using a web-based calculator at http://www.openepi.com. We assumed the prevalence of *Bsm*I homozygous recessives in the control group was 14%; the OR for the CRC group versus the control group was 2.7, based on a study by Rasool et al. [21]. Using Fleiss's method, enrolling 184 patients in each group would provide 80% power to detect this minimum odds ratio at a 1% significance level (a Bonferroni adjusted alpha with 5 SNP comparisons).

## 3. Results

### 3.1. Clinical Characteristics

A total of 364 participants, including 182 with CRC (cases) and 182 without CRC (controls), were included in this study. The mean age of participants in the case and control groups was 62 ± 11 and 62 ± 9 years, respectively, which was not significantly different (*p* = 1.0). The case group had a significantly higher proportion of males than the control group (103 [57%] vs. 59 [32%], *p* < 0.001). Baseline characteristics such as age, history of CRC in first-degree relatives, and BMI were not significantly different between both groups. The mean vitamin D levels were also not significantly different between the case and control groups (24.6 ± 9.1 vs. 25.3 ± 10.6 ng/mL, *p* = 0.52). Moreover, vitamin D deficiency/insufficiency was detected in 76.1% and 79.9% in the case and control groups, respectively, but there was no significant difference. The baseline characteristics of the participants are summarized in Table 1.

### 3.2. Allele Frequencies of *VDR* SNPs

**Figure 1:**
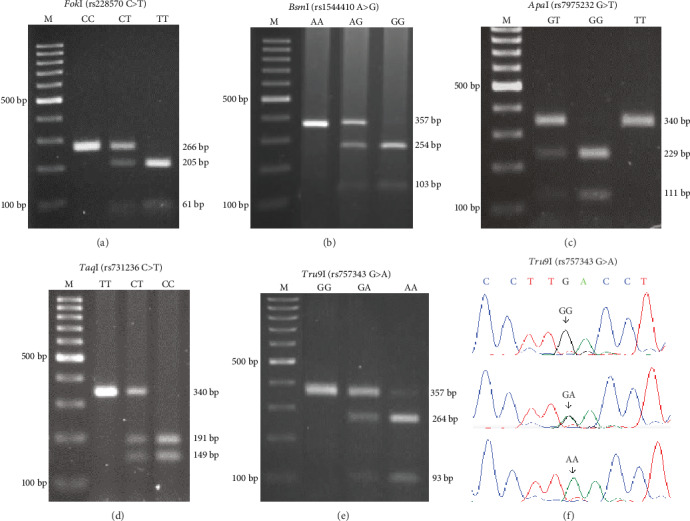
Representative RFLP patterns of each VDR polymorphism of the 5 VDR-SNPs and example of DNA sequencing results of three PCR products of three cases with different *Tru9*I genotypes. (a) *Fok*I (rs228570 C>T): product sizes were 266 bp for the C allele and 205 and 61 bp for the T allele, (b) *Bsm*I (rs1544410 A>G): product sizes were 357 bp for the A allele and 254 and 103 bp for the G allele, (c) *Apa*I (rs7975232 G>T): product sizes were 340 bp for the T allele and 229 and 111 bp for the G allele, (d) *Taq*I (rs731236 C>T): product sizes were 340 bp for the T allele and 191 and 149 bp for the C allele, (e) *Tru9*I (rs757343 G>A): product sizes were 357 bp for the G allele and 26 and 93 bp for the A allele, and (f) DNA sequencing results of the PCR products with *Tru9*I (rs757343 G>A) genotypes GG, GA, and AA. The arrow indicates the polymorphism; M: a molecular marker of the 100 bp ladder.

The case and control groups showed similar proportions of allele frequencies for each *VDR* SNP, and thus, no significant difference in allelic frequencies was found between both groups. The major allele frequencies for each polymorphism are shown in Table 2. The representative PCR-RFLP and the respective sequences of all SNPs analysed are demonstrated in Figure 1.

### 3.3. Genotype Frequencies of *VDR* SNPs

For all *VDR* SNPs, the genotype frequencies were in line with the HWE. Most of the participants in both the case and control groups had the CT genotype, GG genotype, GG genotype, GT genotype, and TT genotype for *Fok*I, *Bsm*I, *Tru9*I, *Apa*I, and *Taq*I, respectively. However, no significant difference in genotype frequency was observed when the case group was compared with the control group (Table 2). The subanalysis comparing vitamin D levels according to genotypic variances in the SNPs showed no significant differences between CRC and control groups (Table 3).

### 3.4. Haplotype Frequencies of *VDR* SNPs

**Figure 2:**
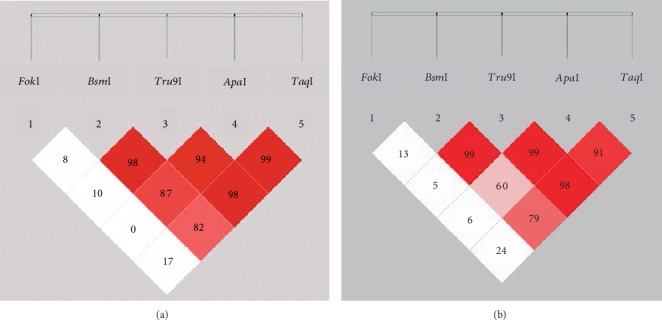
Genomic organisation and linkage disequilibrium (LD) mapping of five common single-nucleotide polymorphisms within the vitamin D receptor (*VDR*) gene in colorectal cancer cases (a) and healthy controls (b). The number and shade of colour in each box represent the LD value in percentage and the strength of the LD, respectively.

A strong LD (*D*′ > 0.8) was observed among *Bsm*I, *Tru9*I, *Apa*I, and *Taq*I (Figure 2). The distribution of the *Bsm*I/*Tru9*I/*Apa*I/*Taq*I haplotype frequencies in the case and control groups is shown in Table 4. The GGGT haplotype was the most frequent haplotype in both the cases and controls (65.8% and 61.5%, respectively). Importantly, the AGGT haplotype was significantly associated with a decreased risk of CRC (odds ratio 0.24, 95% confidence interval 0.07-0.81, *p* = 0.01), whereas other haplotypes did not show any significant difference in frequencies between the cases and controls. Eleven patients with a definite AGGT haplotype were identified, two in the case group and nine in the control group. The vitamin D level was normal in three cases (27.3%), and there was vitamin D insufficiency in five cases (45.4%) and vitamin D deficiency in three cases (27.3%). The detailed data is submitted in Supplementary Table 2.

## 4. Discussion

Most epidemiological and scientific studies have strongly suggested that vitamin D and the *VDR* gene may have a role in colorectal pathogenesis [22, 23] and that variations in *VDR* SNPs are also associated with CRC [19]. This case-control study demonstrated no significant differences in the serum vitamin D levels between CRC cases and controls in the Thai population, as well as no association of the five common *VDR* SNPs, with CRC risk. However, a specific haplotype, AGGT, significantly predicted a lower risk of CRC.

Many studies have attempted to prove the association of common SNPs, including *Fok*I, *Bsm*I, *Tru9*I, *Apa*I, and *Taq*I, with CRC in various populations. A meta-analysis in 2011 including 17 published studies suggested that *Bsm*I is associated with a lower risk of CRC. However, only two studies were conducted in Asian countries, and these studies showed that *Bsm*I and *Fok*I were associated with CRC risk in China and Korea, respectively [24, 25]. A meta-analysis in 2017 by a Chinese study group, which analysed 39 studies worldwide, 9 of which were from Asian countries, showed a strong association between *Bsm*I and CRC risk and a probable but not statistically proven association between *Fok*I and CRC risk [15]. Our study in the Thai population did not reveal any association of the five common SNPs with CRC. The number of cases in each arm of our study was based on a study from Kashmir showing an increased risk (with 2.7 relative risk) of CRC with the *Bsm*I genotype [21]. However, our study not only showed an absence of association between SNPs and CRC but also showed a difference between the *Bsm*I genotypes of our population and that of the study from Kashmir [21]. Such difference due to ethnicity, even within the same geographic area, might help explain the null results in our study. Our results are consistent with those of a large genetic association study of >1700 sibships with and without CRC from North America, Honolulu, and Australia, which clearly showed no statistical association between CRC and 43 *VDR* SNPs, including *Bsm*I and *Taq*I, but not *Tru9*I, *Apa*I, and *Fok*I [26].

In this study, the analysis of the haplotype AGGT of *Bsm*I, *Tru9*I, *Apa*I, and *Taq*I showed statistically significant results in relation to CRC. However, the association was quite minimal, with allele frequencies of only 0.9% and 3.6% in the case and control arms, compared to that of genetic association studies. The significant association from our haplotype analysis was only based on a number of cases initially calculated to prove an association between CRC and a single SNP, specifically *Bsm*I. Thus, to prove the association between the AGGT haplotype and CRC in our population, the next study will require at least >1000 cases in each arm. Several case-control studies found some specific haplotypes that are associated with CRC. A case-control study in the USA with >3000 White, Hispanic, African-American, and Asian cases and controls examined the haplotypes of three SNPs spanning across the *VDR* gene, including *Bsm*I (intron 8), poly(A) long (18-22 repeats), and *Fok*I (exon 2), and reported an association of the bLF and BLF haplotypes with the risk of colon cancer [27]. Other studies showed a significant association of *Bsm*I/*Apa*I/*Taq*I and *Bsm*I/*Taq*I haplotypes with a decreased risk of CRC in Korean and European populations, respectively [25, 28]. However, our study demonstrated that in the Thai population, all SNPs did not deviate from the HWE, and that *Bsm*I, *Tru9*I, and *Apa*I in intron 8 and *Taq*I in exon 9 were more likely to have low recombination rates and be inherited together, with *Bsm*I and *Apa*I showing high and moderate LD scores only in the control group. The importance of the HWE and LD in genetic association studies has been reviewed and should be assessed for this type of studies [29, 30]. In our study, we found that the LD of the *Bsm*I/*Tru9*I/*Apa*I/*Taq*I haplotype was within block B, spanning approximately 40 kilobases (kb) and containing exons 3-9, of the three LD block patterns reported in European studies, which showed that these patterns are likely to be inherited together even in different European ethnicities [30].

The association between CRC and these specific haplotypes from different populations, including ours, points to the same hypothesis that some specific variations in the genome, likely in the *VDR* gene or possibly in a nearby gene, affect the VDR functions. The haplotype AGGT in our study is a nonsynonymous variation, which is located on intron 8 and exon 9, nearer to the 3′-UTR. It is unlikely to affect the translated VDR polypeptides [12]. There are several speculations of the effect of these 3′-UTR variations that might affect the transcription ability of the *VDR* gene in osteoblast cell lines [31]. Two of the most important genetic tools to elucidate *in vivo* protein-gene interactions that have been recently developed are chromatin immunoprecipitation (ChIP) and ChIP with massively parallel sequencing (ChIP-seq) [32, 33]. These methods open new insights not only into the vitamin D response element (VDRE), which is located in the promoter region of hundreds of genes involved in different cellular functions, but also into the biology of VDR, especially the transcriptional activation of the *VDR* gene. Importantly, the VDR has its own autoregulation through several enhancer elements far upstream from the transcription start site and also inside the *VDR* gene, reported to be 20-30 kb downstream inside the introns [34, 35]. The haplotype associated with CRC in our and other studies might be linked to these enhancer elements; however, further study is required to prove this hypothesis. The VDR has low expression in intestinal epithelial cells. Enhanced VDR-mediated transcripts in stromal fibroblasts surrounding cancer cells could predict better survival outcomes [36]. Hence, this specific haplotype associated with a decreased CRC risk in the Thai population might represent or be associated with the enhancer variants inside the *VDR* intron.

Several epidemiological studies, mostly from Western countries, including meta-analyses, affirm the link between lower vitamin D levels and a higher risk of CRC [22, 37]. In other words, adequate vitamin D levels protect against CRC development [37]. In this study, even though the population was from the tropical regions with high sunlight exposure throughout the year, nearly 80% of the participants from the case and control groups had vitamin D levels below 30 ng/mL, and one-third of the participants from the case group had vitamin D levels below 20 ng/mL or had vitamin insufficiency/deficiency. Moreover, participants in both the case and control groups showed no difference in vitamin D levels. We were able to identify 11 patients with the AGGT haplotype, and the vitamin D levels were normal in three cases (27.3%), insufficient in five cases (45.4%), and deficient in three cases (27.3%). These findings could be attributed to the lack of association between the haplotype and vitamin D levels, as nearly three quarters of the patients had vitamin D levels below 30 ng/mL; however, a larger sample size is required to prove this assumption. In addition, these serum vitamin D levels do not always reflect its local tissue levels and the magnitude of *VDR* gene activation in different haplotype variants.

This study has some limitations. As mentioned earlier, the number of cases is quite small, calculated only to prove the association between CRC and a single SNP, and the analysis was based on data obtained from another Asian study [21]. The haplotype analysis also requires a second larger study to confirm the results. Furthermore, the vitamin D levels measured in this study were cross-sectional results and therefore cannot represent long-term levels to reflect patient lifestyles and several other factors. Lastly, we could not entirely prove that low vitamin D levels can influence the risk of CRC in the Thai population, as reported in other studies [37].

## 5. Conclusions

In conclusion, this study revealed that it remains difficult to confirm the association of low vitamin D levels and *VDR* gene variations with colorectal carcinogenesis in the Thai population. The five SNPs, namely, *Fok*I, *Bsm*I, *Tru9*I, *Apa*I, and *Taq*I, were not associated with CRC. Interestingly though, a specific haplotype, AGGT, of the *Bsm*I, *Tru9*I, *Apa*I, and *Taq*I SNPs was associated with a decreased risk of CRC, but further study with a larger sample is required to confirm such association. Our study indicates that further research should not focus on the common *VDR* SNPs but should explore some recently discovered enhancer elements associated with CRC.

## Figures and Tables

**Table 1:** Comparison of clinical characteristics between colorectal cancer cases and controls.

Characteristic	CRC cases *n* = 182	Controls *n* = 182	*p* value
Age (years)	62 ± 11	62 ± 9	1.00
Male, *n* (%)	103 (57%)	59 (32%)	<0.001
History of CRC in 1st-degree relatives, *n* (%)	128 (70%)	134 (74%)	0.48
Body mass index (kg/m^2^)	23 ± 6	24 ± 4	0.06
	*n* = 155	*n* = 174	
Vitamin D level (ng/mL)	24.6 ± 9.1	25.3 ± 10.6	0.52
Vitamin D status, *n* (%)			
(i) Severe deficiency (<10 ng/mL)	7 (4.5%)	3 (1.7%)	0.30
(ii) Deficiency (10-19.99 ng/mL)	40 (25.8%)	43 (24.7%)	
(iii) Insufficiency (20-29.99 ng/mL)	71 (45.8%)	93 (53.4%)	
(iv) Normal (≥30 ng/mL)	37 (23.9%)	35 (20.1%)	

CRC: colorectal cancer.

**Table 2:** Genotype and allele frequencies of *VDR* SNPs in cases and controls.

SNPs	Allele	Frequency (%)		*p* value	OR (95% CI)	Genotype	Frequency (%)		*p* value	*p* ^HWE^
		Cases	Controls				Cases	Controls		
*Fok*I	C	50.3	47.5	0.46	1.12	C/C	23.1	25.3	0.13	0.15
	T	49.7	52.5		(0.83-1.49)	C/T	54.4	44.5		
						T/T	22.5	30.2		
*Bsm*I	A	8.5	12.1	0.11	0.68	A/A	0.5	2.2	0.24	0.35
	G	91.5	87.9		(0.41-1.09)	A/G	15.9	19.8		
						G/G	83.5	78.0		
*Tru9*I	A	25.0	24.7	0.93	1.01	A/A	6.6	4.9	0.73	0.40
	G	75.0	75.3		(0.72-1.40)	A/G	36.8	39.6		
						G/G	56.6	55.5		
*Apa*I	G	67.6	65.4	0.53	1.1	G/G	45.1	43.4	0.71	0.70
	T	32.4	34.6		(0.81-1.50)	G/T	45.1	44.0		
						T/T	9.9	12.6		
*Taq*I	C	5.5	6.6	0.53	0.82	C/C	0.0	1.1	0.37	0.15
	T	94.5	93.4		(0.45-1.52)	C/T	11.0	11.0		
						T/T	89.0	87.9		

CI: confidence interval; OR: odds ratio; *p*^HWE^: *p* values of the Hardy-Weinberg equilibrium.

**Table 3:** Vitamin D levels according to SNP genotypes in the patients with CRC compared to the control groups.

SNPs	Genotypes (*n* CRC, control)	Vitamin D level (mean ± SD)		*p* value
		CRC	Control	
*Fok*I	CC (33, 42)	22.3 ± 9.6	25.6 ± 10.0	0.156
	CT (87, 81)	24.7 ± 9.0	23.9 ± 7.6	0.518
	TT (35, 51)	26.6 ± 8.7	27.3 ± 14.3	0.798

*Bsm*I	AA (1, 3)	21.4	21.4 ± 5.7	0.996
	AG (25, 31)	26.8 ± 8.5	24.6 ± 8.2	0.342
	GG (129, 140)	24.3 ± 9.3	25.5 ± 11.1	0.304

*Tru9*I	AA (12, 9)	30.1 ± 15.9	33.6 ± 23.0	0.684
	AG (62, 68)	23.3 ± 8.5	23.9 ± 7.3	0.616
	GG (81, 97)	24.9 ± 8.1	25.5 ± 10.6	0.686

*Apa*I	GG (65, 78)	24.7 ± 8.0	26.2 ± 11.4	0.371
	GT (72, 75)	24.4 ± 9.6	23.7 ± 7.2	0.62

**Table 4:** Allele frequencies of *Bsm*I/*Tru9*I/*Apa*I/*Taq*I haplotypes.

*Bsm*I/*Tru9*I/*Apa*I/*Taq*I haplotype	Frequency (%)		*p* value	OR	95% CI
	Cases	Controls			
*AATT*	0.5	0.0	0.31	32.22	1.78-607.92
*AGGT*	0.9	3.6	0.01	0.24	0.07-0.81
*AGTC*	4.3	5.5	0.45	0.77	00.39-1.52
*AGTT*	2.7	3.0	0.78	0.89	0.37-2.12
*GATT*	23.4	24.7	0.67	0.93	0.66-1.30
*GGGC*	0.0	0.3	0.31	—	—
*GGGT*	65.8	61.5	0.23	1.20	0.89-1.63
*GGTC*	1.0	0.8	0.82	1.20	0.26-5.48
*GGTT*	0.4	0.6	0.73	0.67	0.08-5.84
*AATC*	0.2	0.0	0.38	—	—
*GAGT*	0.9	0.0	0.07	—	—

CI: confidence interval; OR: odds ratio.

## Data Availability

The genotype data of participants were submitted in Dryad (identifier number: 336 doi:10.5061/dryad.6hdr7sqx0).

## Supplementary Materials

Supplementary 1 Supplementary Table 1: primer sequences and PCR-RFLP analysis.

Supplementary 2 Supplementary Table 2: vitamin D levels in eleven patients with a definite AGGT haplotype among CRC and control groups.
