# Supplementary material for: Association between Vitamin D Receptor Single-Nucleotide Polymorphisms and Colorectal Cancer in the Thai Population: A Case-Control Study
Source: Biomed Res Int. 2020 Jun 15;2020:7562958. doi: 10.1155/2020/7562958 (PMC7313039; doi:10.1155/2020/7562958)
Supplement: Supplementary 1 — Supplementary Table 1: primer sequences and PCR-RFLP analysis. [file 7562958.f1.docx]

**Supplementary Table 1** Primer sequences and PCR-RFLP analysis

| **SNPs** | **Primer sequence** | **Restriction enzyme** | **Allele** | **Product size, bp** |
| --- | --- | --- | --- | --- |
| **rs2228570** | F: 5' TGG CAC TGA CTC TGG CTC TGA 3' | *Fok*I | T | 205, 61 |
|  | R: 5' CTC CCT TCA TGG AAA CAC CTT G 3' |  | C | 266 |
| **rs1544410** | F: 5' CTC ACT GCC CTT AGC TCT GC 3' | *Bsm*I | G | 254, 103 |
|  | R: 5' TTG GAC CTC ATC ACC GAC AT 3' |  | A | 357 |
| **rs757343** | F: 5' CTC ACT GCC CTT AGC TCT GC 3' | *Tru*9I | A | 264, 94 |
|  | R: 5' TTG GAC CTC ATC ACC GAC AT 3' |  | G | 357 |
| **rs7975232** | F: 5' TTT GGG GCC AGG CAG TGG TAT 3' | *Apa*I | G | 229, 111 |
|  | R: 5' CGG TAC TGC TTG GAG TGC TCC TC 3' |  | T | 340 |
| **rs731236** | F: 5' TTT GGG GCC AGG CAG TGG TAT 3' | *Taq*I | C | 191, 149 |
|  | R: 5' CGG TAC TGC TTG GAG TGC TCC TC 3' |  | T | 340 |
