# Supplementary material for: Association between Vitamin D Receptor Single-Nucleotide Polymorphisms and Colorectal Cancer in the Thai Population: A Case-Control Study
Source: Biomed Res Int. 2020 Jun 15;2020:7562958. doi: 10.1155/2020/7562958 (PMC7313039; doi:10.1155/2020/7562958)
Supplement: Supplementary 2 — Supplementary Table 2: vitamin D levels in eleven patients with a definite AGGT haplotype among CRC and control groups. [file 7562958.f2.docx]

**Supplementary Table 2** Vitamin D levels in eleven patients with a definite AGGT haplotype among CRC and control groups

| Case number | VDR SNPs’ genotypes | | | | Diagnosis | Vitamin D  levels (ng/mL) |
| --- | --- | --- | --- | --- | --- | --- |
|  | *Bsm*I | *Tru9*I | *Apa*I | *Taq*I |  |  |
| 1 | GA | GG | GG | TT | CRC | 19.7 |
| 2 | GA | GG | GG | TT | CRC | 34.3 |
| 3 | GA | GG | GG | TT | Control | 14.4 |
| 4 | GA | GG | GG | TT | Control | 18.5 |
| 5 | GA | GG | GG | TT | Control | 20.7 |
| 6 | GA | GG | GG | TT | Control | 21.7 |
| 7 | GA | GG | GG | TT | Control | 22.1 |
| 8 | GA | GG | GG | TT | Control | 26.1 |
| 9 | GA | GG | GG | TT | Control | 29.5 |
| 10 | GA | GG | GG | TT | Control | 32.3 |
| 11 | GA | GG | GG | TT | Control | 57.4 |
